# The Human Central Canal of the Spinal Cord: A Comprehensive Review of its Anatomy, Embryology, Molecular Development, Variants, and Pathology

**DOI:** 10.7759/cureus.927

**Published:** 2016-12-12

**Authors:** Erfanul Saker, Brandon M Henry, Krzysztof A Tomaszewski, Marios Loukas, Joe Iwanaga, Rod J Oskouian, R. Shane Tubbs

**Affiliations:** 1 Department of Anatomical Sciences, St. George's University School of Medicine, Grenada, West Indies; 2 Department of Anatomy, Jagiellonian University Medical College, Krakow, Poland; 3 Seattle Science Foundation; 4 Neurosurgery, Complex Spine, Swedish Neuroscience Institute; 5 Neurosurgery, Seattle Science Foundation

**Keywords:** anatomy, spine, spinal cord, central canal, syringomyelia

## Abstract

The human central canal of the spinal cord is often overlooked. However, with advancements in imaging quality, this structure can be visualized in more detail than ever before. Therefore, a timely review of this part of the cord seemed warranted. Using standard search engines, a literature review was performed for the development, anatomy, and pathology involving the central canal. Clinicians who treat patients with issues near the spine or interpret imaging of the spinal cord should be familiar with the morphology and variants of the central canal.

## Introduction and background

The human central canal extends throughout the spinal cord [1-2]. Derived from the primitive neural tube, the central canal encompasses an internal system of cerebrospinal fluid (CSF) cavities that include the cerebral ventricles, aqueduct of Sylvius, and fourth ventricle [1]. Uniformly elliptical in shape throughout most of the cord, variations exist in the morphology of the caudal central canal, including dilatation [3-4], forking [5-6], and outpouchings [6-7]. The function of the adult human central canal is not yet well understood [7]. Often regarded as vestigial and a structure that is obliterated after birth and replaced by ependymal cells, the canal may be patent up to the second decade of life. Pathology involving the central canal includes stenosis or occlusion, hydrosyringomyelia, and other cavitary lesions [1, 7]. It is difficult to discuss these lesions without a better understanding of the canal. Therefore, the objective of this review is to elaborate further on our current understanding of the anatomy and importance of the human central canal of the spinal cord. 

## Review

### Embryology

The central canal arises from the neural canal, formed within the neural tube during neurulation. During the fourth week (22-23 days) of development, in the region of the fourth to sixth pairs of somites, the underlying notochord and paraxial mesoderm induce the overlying ectoderm to differentiate into the neural plate [8]. The neural tube and the neural crest differentiate from the neural plate. Signaling molecules involved in this process include the transforming growth factor-β (TGF-β) family, which includes activin and fibroblast growth factors (FGFs) [8].

**Figure 1:**
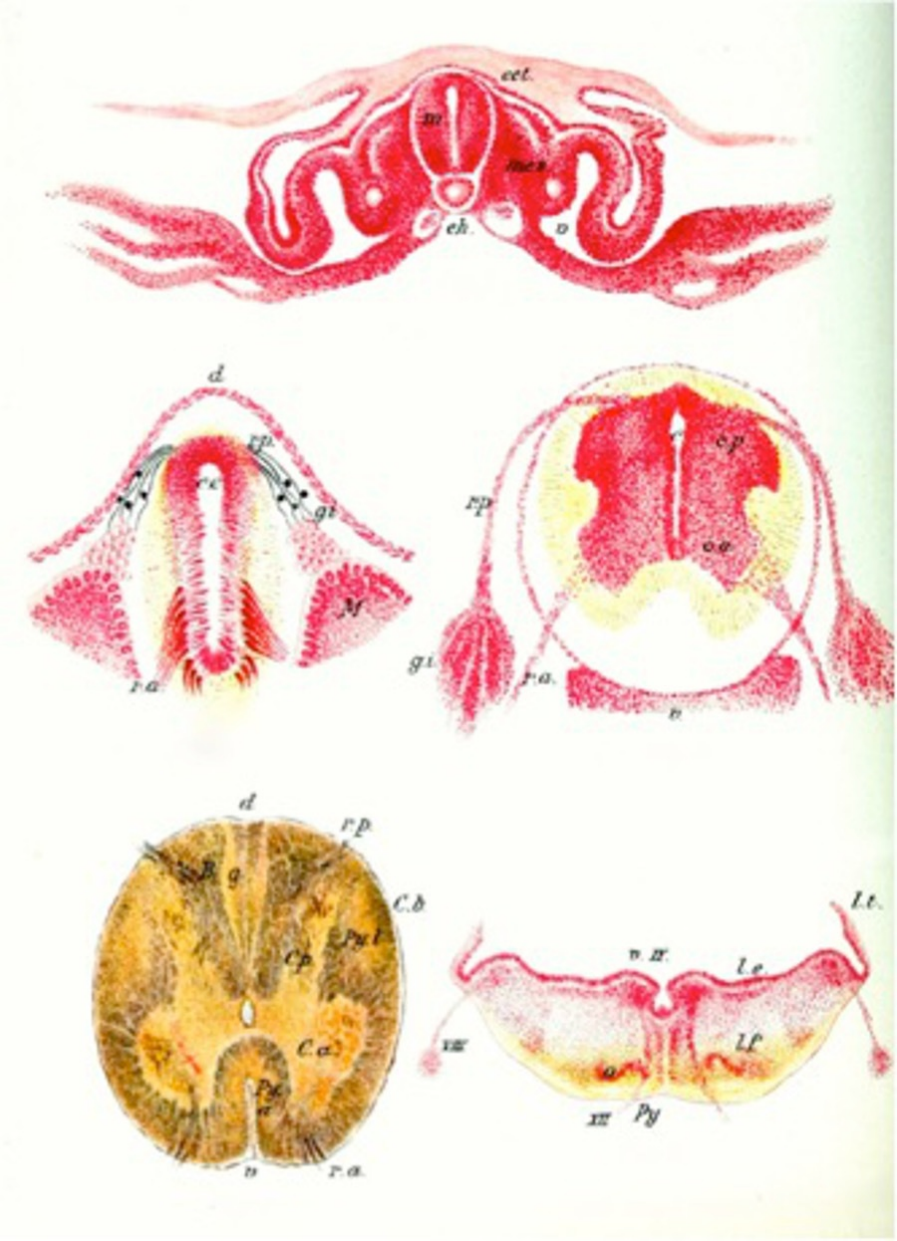
Various embryological stages of the neural tube noting the evolution of the central canal to its final position in the adult spinal cord (lower left image). (From Jacob’s Atlas of the Nervous System, 1901)

In the early part of the fourth week, the caudal one-third of the neural plate (caudal to the fourth pair of somites) and neural tube represent the future spinal cord (Figure 1). Fusion of the neural folds occurs in a cranial to caudal direction until only small areas of the neural tube remain open at both ends, with its lumen (neural canal) communicating freely with the amniotic cavity [8]. The cranial opening, the rostral neuropore, closes around day 25, and the caudal neuropore closes on day 27 at which point the neural canal is converted into the ventricular system of the brain and the central canal of the spinal cord. The lateral walls of the neural tube thicken while the dorsal and ventral parts remain thin and are named the roof and floor plates [9]. Thickening of the tube gradually reduces the size of the neural canal until only a minute central canal of the spinal cord is present at nine to 10 weeks [8].

**Figure 2:**
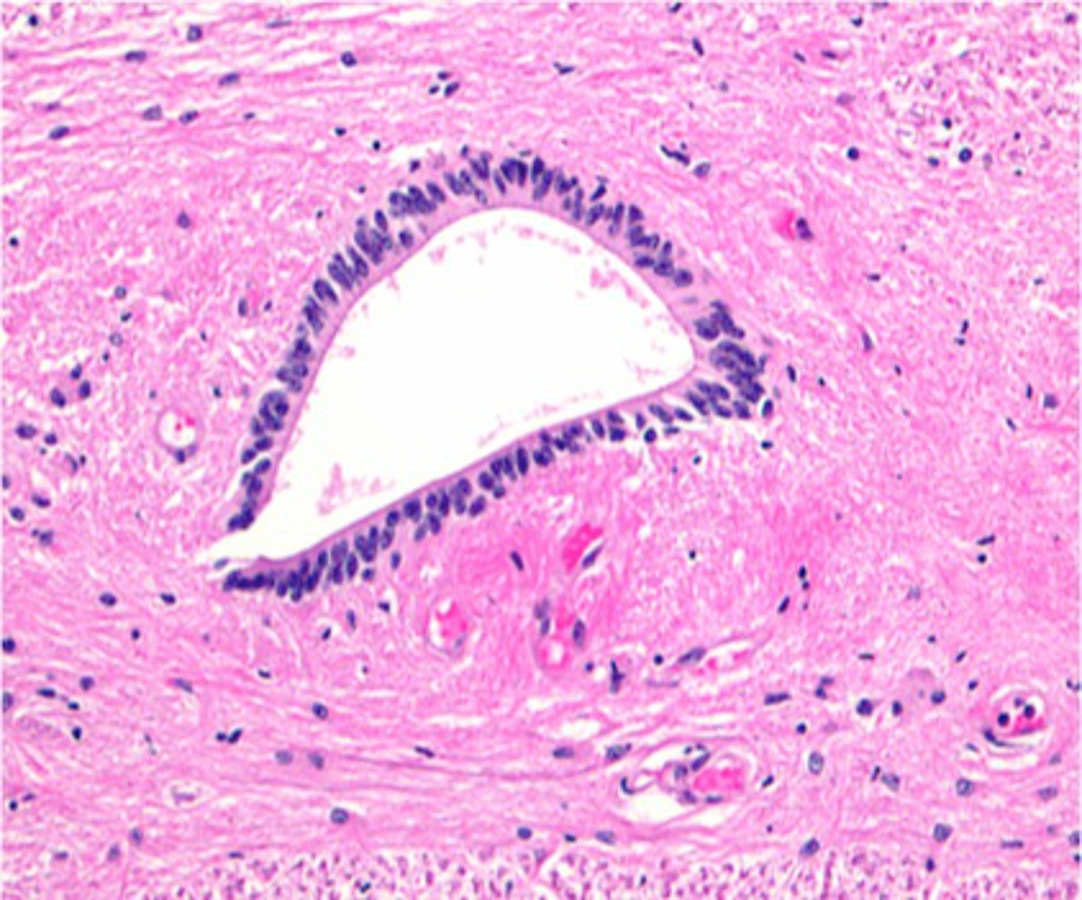
Histological image (H&E) of the human central canal at the center of the image. Note the single layer of columnar ependymal cells.

The wall of the neural tube is composed of a single layer of pseudostratified, columnar neuroepithelium that constitutes the ventricular zone (ependymal layer) and gives rise to all neurons and macroglial cells (astroglia and oligodendroglia) in the spinal cord (Figure 2) [8]. The neuroepithelial cells in the ventricular zone differentiate into neuroblasts, which give rise to neurons. When neuroblast formation ceases, the neuroepithelial cells differentiate into glioblasts (spongioblasts). These cells migrate from the ventricular zone into other zones formed from components of the neuroepithelial cells. Some of the glioblasts become astroblasts and then astroglia (astrocytes). Others become oligodendroblasts and then oligodendroglia (oligodendrocytes). The remaining neuroepithelial cells differentiate into ependymal cells lining the central canal of the spinal cord [8-9]. 

### Anatomy

The central canal, also referred to as the spinal foramen or ependymal canal, extends from the conus medullaris in the lumbar spine to the caudal angle of the fourth ventricle and is lined by a single layer of columnar ependymal cells [2]. It represents the remnant of the lumen of the primitive neural tube. As one ages, the canal fills with the disintegrated cellular debris of the lining epithelium [10-12].

**Figure 3:**
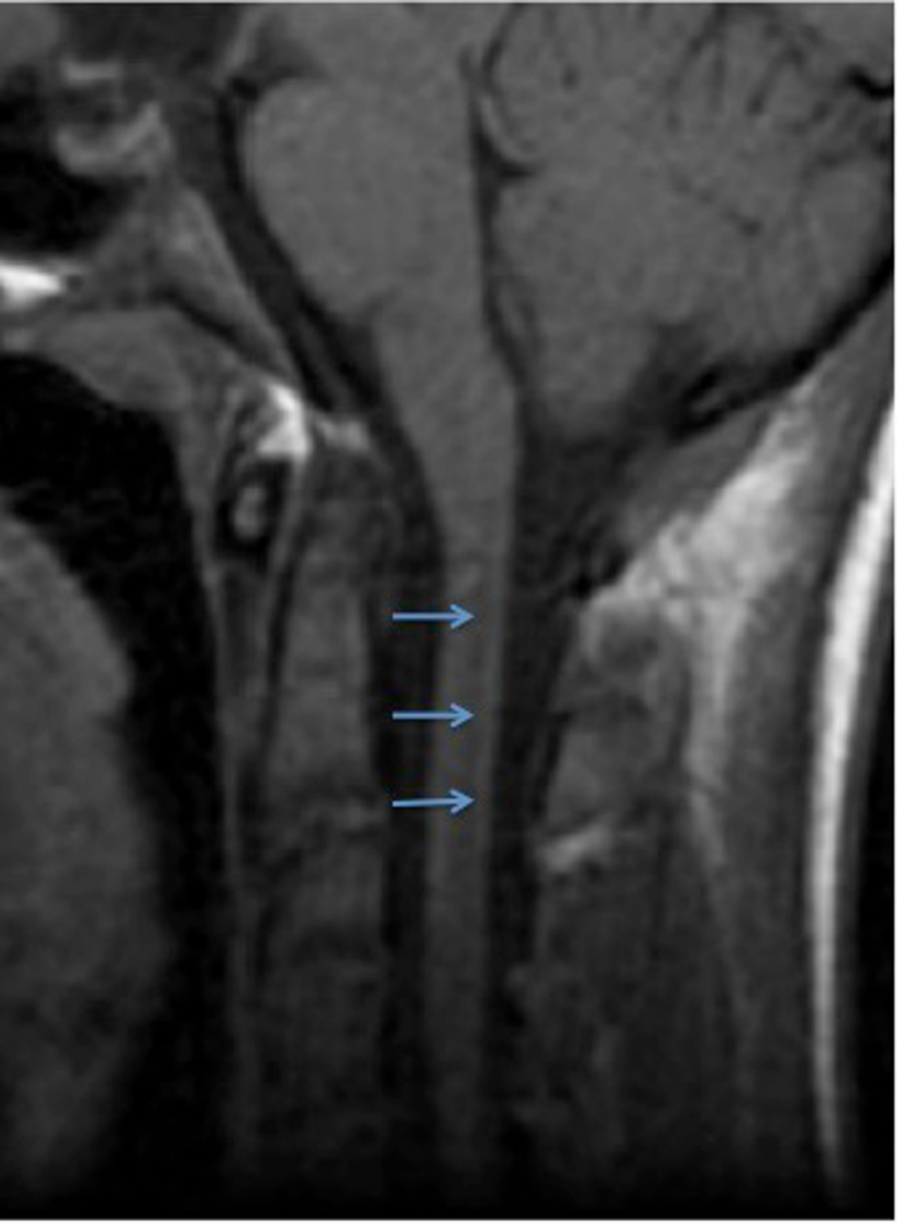
T1-weighted sagittal MRI noting a normal central canal (arrows).

**Figure 4:**
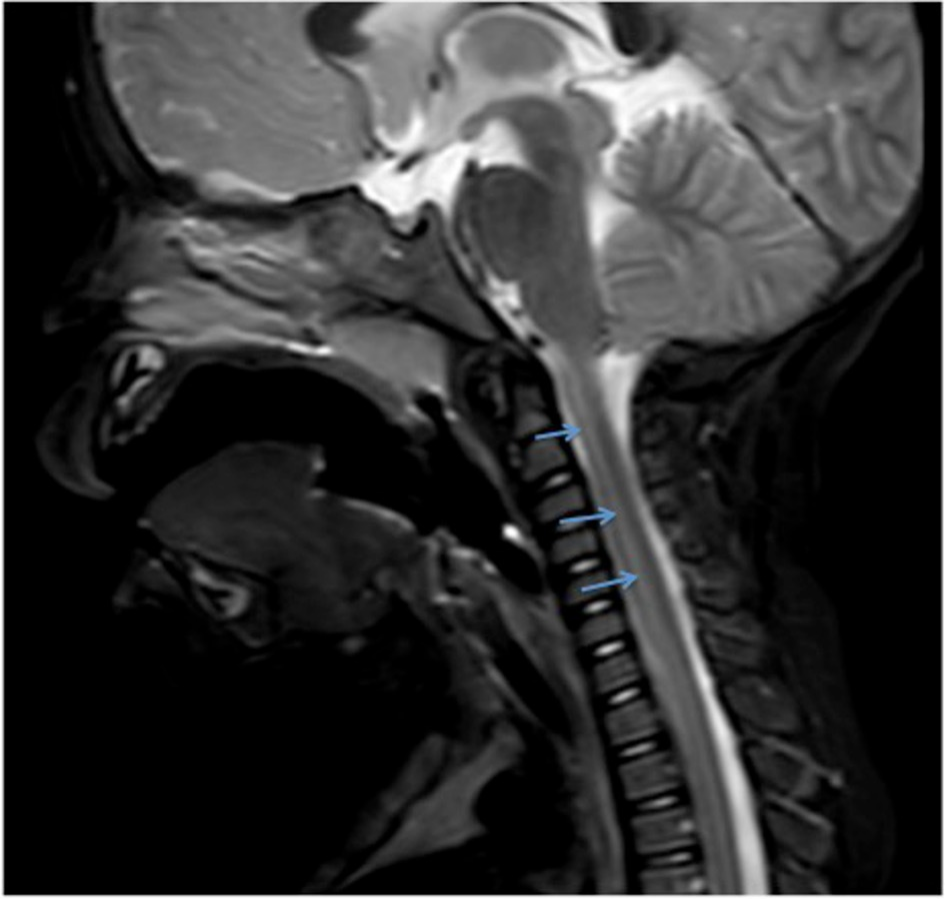
T2-weighted sagittal MRI noting a normal central canal (arrows).

**Figure 5:**
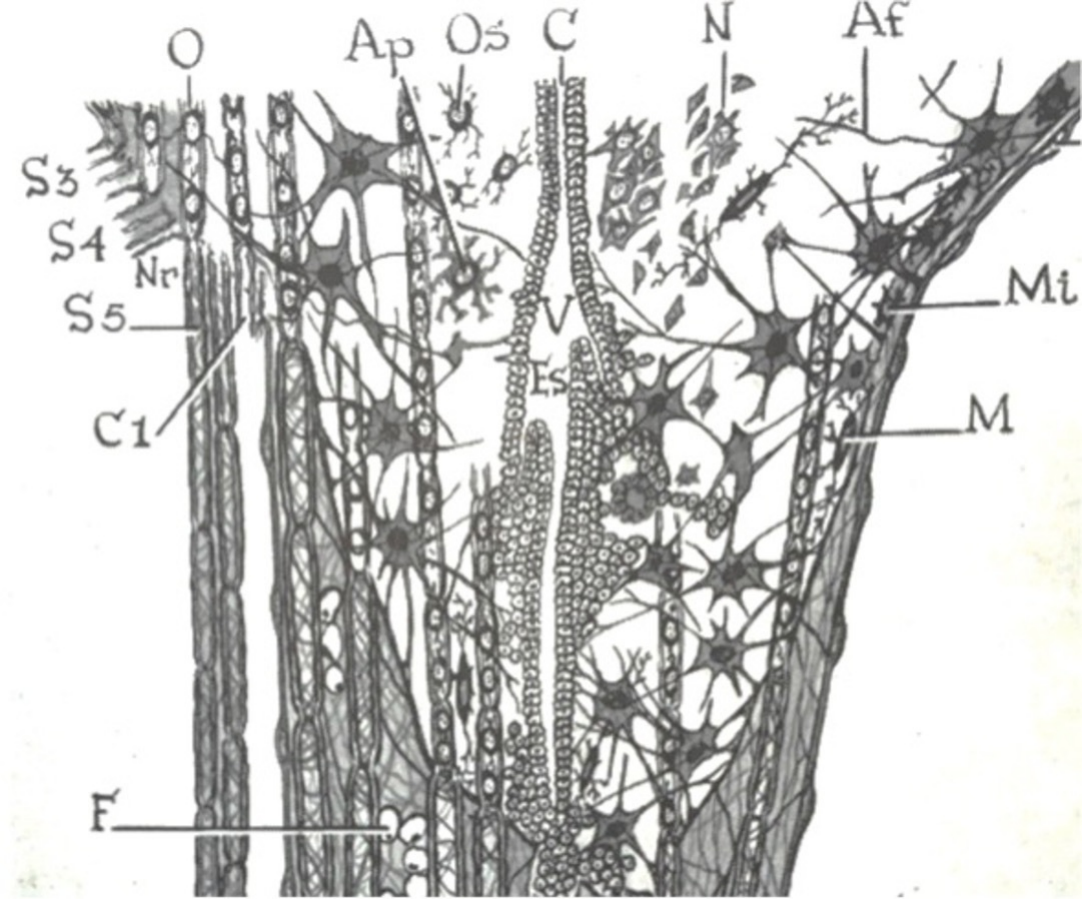
Schematic drawing of the distal conus medullaris of the spinal cord in the coronal plane noting the central canal (C) and its dilatation into the terminal ventricle (V). Interfascicular oligodendrocytes (O), protoplasmic astrocyte (Ap), nerve cells (N), and microglia cells (M) (From Tarlov’s 1953 Sacral Nerve Root Cysts)

The central canal is part of a system of cerebrospinal fluid (CSF) cavities that includes the cerebral ventricle, aqueduct of Sylvius, and fourth ventricle (Figures 3-4) [2]. It is situated in the gray commissure, which (along with the anterior white commissure) connects the two parts of the spinal cord. The gray commissure can be divided into dorsal and ventral components, based on their relationship to the central canal [10]. As the spinal cord merges into the medulla, the canal trends backward and opens into the fourth ventricle. In the conus medullaris, it is more dorsally located, becomes widened, and forms the triangular-shaped, 8-to-10 mm long structure known as the ventriculus terminalis of Krause (Figure 5) [10-11].

The location of the central canal relative to the midpoint of the spinal cord varies among the regions of the cord. It is slightly ventral in the cervical and thoracic segments, central in the segments of the lumbar region, and dorsal in the conus medullaris [10]. In transverse sections, the central canal appears morphologically oval or circular and is juxtaposed by the substantia gelatinosa centralis of Stilling, which contains neurons, neuroglia, and a reticulum of fibers [10-11]. The central canal is also lined with cylindrical epithelium, which bears cilia in the embryonic cord. The epithelial cells have basal processes, which are continuous with the neuroglial tissue upon which they rest [11].

The region of the central canal is rich in neuroglia cells and fibers. These are chiefly arranged in the form of a circular network just beneath and around the central canal. In front and behind the central canal, the fibers display a commissural-like arrangement; laterally, they are continuous with the fibers of the anterior horns [11].

### Variations 

The central canal is usually a uniform elliptical [13] shape throughout most of the cord, although variations in the morphology of the caudal central canal have been described [7]. Choi, et al. noted that the ependyma-lined central canal of the filum terminale forms a cystic dilatation at the lower end of the conus medullaris to become the ventriculus terminalis of the spinal cord [3]. Similarly, enlargement of the canal was reported by Pearson and Sautter in their observations on the caudal end of the spinal cord [4]. They reported the gray matter of the alar plate was reduced in size with the corresponding enlargement of the central canal, which constituted the terminal ventricle. Multiple cases of canal duplication in human embryos, and whether they are of primary or secondary origin have been described in the literature [5, 14-16].

Ikeda inspected the caudal end of the nerve cord in human embryos and found multiple cases with forking of the cord lumen, reporting seven main types [5]. Similarly, Lendon and Emery investigated the incidence of canal forking in the equinal cord of 100 human infants [6]. They observed that most of the forking occurred at the caudal end of the region, particularly in the filum terminale, surmising that the process of redifferentiation associated with the development of the filum contributed to the incidence of canal forking [6]. Storer, et al. performed a computerized 3-D study of the central canal and observed forking in the lower part of the conus near the terminal ventricle with outpouchings of each fork of the central canal into the filum terminale [7]. Additionally, they observed the proliferation of ependymal cells in two distinct columns of the lower conus and upper filum with the extension of these cells from the lumen of the canal to the surface of the pia mater. They speculated this was a possible functional connection between the canal and the subarachnoid space providing an important fluid communication that may play a role in the “sink” function of the canal [10].

Studies of various species, including some primates and German shepherd dogs [17], have demonstrated openings from the filum terminale central canal into the subarachnoid space [7, 18-22].

### Pathology 

Syringomyelia

Typically, the central canal is prone to certain types of diseases, destructive lesions, and conditions that become more common as people age. The central canal is frequently involved with cavitary lesions in the parenchyma of the spinal cord, such as syringomyelia [10]. First introduced by Ollivier D’Angers in 1827, the term syringomyelia, derived from the Greek word for tube (syrinx), is used to describe dilation of the central canal extending over many segments and appears to be related to a hydrodynamic mechanism related to the cerebrospinal fluid (CSF) [10, 23].

**Figure 6:**
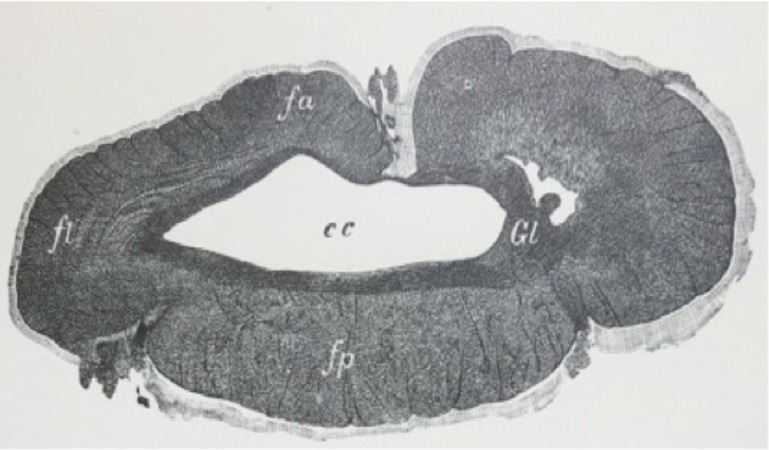
Axial cut through the spinal cord from a patient with dilatation of the central canal (cc), i.e., hydrosyringomyelia.

Often used interchangeably in literature with syringomyelia, the closely related term hydromyelia also refers to a dilatation of the central canal by CSF (Figure 6). Some have defined hydromyelia as a congenital dilatation [24] of the central canal associated with hydrocephalus, an obstruction of the foramina of Luschka and Magendie [25], and is at least partially lined by ependymal cells. Syringomyelia has been applied to every kind of intramedullary cyst by some authors defining it as a cavity distinct from the central canal and lined by ependymal cells or primarily glial cells [23, 25]. Others restrict its use to certain subtypes of cystic lesions and distinguish syringomyelia, hydromyelia, or myelomalacia as separate entities. Still, some authors combine these terms into syringohydromyelia or hydrosyringomyelia [25]. Lee, et al. stated that a clear communication between intramedullary cavities and the ventricular system is almost never demonstrated, making it difficult to differentiate syringomyelia from hydromyelia, although a truly eccentric location within the spinal cord may be more characteristic of syringomyelia than of hydromyelia [26]. Batzdorf mentioned the distinction between syringomyelia and hydromyelia is no longer considered absolute or critical [23].

Many have postulated on the pathophysiology of syringomyelia, but it is still not well understood. Gardner in 1958 suggested a “water-hammer theory” where obstruction of the foramen of Magendie leads to the transmission of pulsatile CSF pressure into the central canal through the obex [27]. Ball and Dayan surmised that the CSF enters the syrinx through an enlarged Virchow-Robin space in the spinal cord via a one-way valve-like mechanism at the craniovertebral junction, which blocks the upward CSF movement [28]. Chang and Nakagawa performed the simulation of CSF dynamics and came to the conclusion that loss of the shock absorbing capacity of the cisterna magna and subsequent increase of central canal wall pressure leads to syrinx formation [29]. Studies on kaolin-induced hydrocephalic animals indicated that the forces of a downward movement of CSF from ventricles in the brain into the central canal caused spinal cord cavitation, including central canal distention and disruption into the cord parenchyma [30-37]. Klekamp stated that if we understand syringomyelia as a state of chronic interstitial edema, where the extracellular fluid is trapped in the spinal cord due to CSF flow obstruction, spinal cord tethering, or an intramedullary tumor, then we could explain all of the experimental and clinical observations mentioned throughout literature [25].

Since the anterior white commissure lies near the central canal, it is likely to be the first structure that gives definite clinical signs [38]. Involvement of the anterior horns may lead to amyotrophy, often first evident in or confined to the hands but later spreading to the forearm muscles of the shoulder girdle. Deep reflexes in the upper extremities are diminished early in the course of the disease, resultant of the loss of continuity in the reflex arc. Scoliosis is often an early sign resulting from damage to the dorsomedial and ventrolateral spinal nuclei. The decussating spinothalamic fibers, carrying pain and thermal sensation, are frequently interrupted, leading to impairment and subsequent loss of pain and temperature sensibility with retention of light touch and proprioception [23]. This pathological condition may occur at any level of the spinal cord, but if it should appear at extremity levels, clinical suspicion should be wary for syringomyelia as such patients often seek treatment for burns, cuts, or blisters on the regions involved [38]. 

Central Canal Stenosis

It is estimated that stenosis of the central canal or occlusion occurs in 70% to 80% of normal adults [1]. The canal narrows over time as part of an involutional or degenerative process and compresses the nerves, resulting in claudication and radicular pain in the lower extremities. A distinctive feature of the human central canal is its tendency to become progressively occluded after birth [1]. However, not all levels of the canal become stenotic or obliterated with age. An autopsy study by Yasui, et al. showed portions of the central canal in the cervical cord remain patent up to the fourth to sixth decades of life [2]. Muthukumar was able to report a patent central canal in a 33-year-old patient diagnosed with panventriculomegaly with communicating syringomyelia [39]. Histologic studies of the canal indicate that ependymal cell breakdown during the aging process contributes to the canal occlusion [10]. The ependymal cell changes result in glial bundle formation, the proliferation of astrocytes, formation of subependymal gliovascular buds, and intracanalicular gliosis [1-2, 10]. 

Milhorat, et al. performed a large autopsy study and observed that the stenotic process was most pronounced in the narrowest segments of the canal (T2-T8) and involved more levels with higher grades of stenosis in older individuals [1]. Interestingly, they suggested that central canal stenosis was an acquired pathologic lesion rather than an age-dependent degenerative process [10]. They speculated that the cause is recurring episodes of ependymitis caused by the common virus infections one is exposed to throughout life [10]. This theory is supported by Mims who has shown that influenza A and poxviruses replicate selectively in ependymal cells [40]. Other viruses, including parainfluenza 2, measles, mumps, and reovirus type I, were also shown to infect and destroy ependymal cells in the absence of clinically apparent disease [1, 40]. 

### Molecular studies

Many species have been used to better understand the anatomy and pathological nuances involving the spinal cord central canal. Garcia-Ovejero, et al. [41] were able to demonstrate that the adult human central canal displays at least three common characteristics distinct from other species: first, a gliosis formed by a dense mesh of glial fibrillary acidic protein-positive ( GFAP+) astrocytic processes. A second feature is the presence of protoplasmic cells or ependymocytes, which show expression of CD15 and glutamate aspartate transporter (GLAST) (now known as SLC1A3), that, in addition to astrocytes, have been related to radial glia and stem/precursor cell phenotypes [41]. Additionally, Paniagua-Torija, et al. observed that the ependymal region is enriched in cannabinoid receptor type 1 (CB1), which is involved in the regulation of the neural stem [42]. A third feature is the existence of structures previously described as pseudo-canals consisting of cells expressing vimentin and radially oriented around a presumed lumen, which later was discovered to be a blood vessel [41]. Garcia-Ovejero, et al. stated that this finding showed the presence of perivascular pseudorosettes, a crucial diagnostic feature for low-grade ependymoma [41-42].

Recent adult mammalian spinal cord studies have revealed that ependymal cells lining the central canal retain latent neural stem cell potential following experimental spinal cord injury (SCI) [43-44]. Immunohistochemical studies of the spinal cord ependymal zone revealed ependymal cells bordered by a subependymal layer comprised of small numbers of astrocytes, oligodendrocyte progenitors, and neurons [43]. It was found that the dorsal pole of the central canal contains tanycyte-like cells that express markers of both ependymal cells and neural precursors, suggesting the potential for stem cell activity [43]. Lee, et al. performed real-time polymerase chain reaction (PCR) array analysis of mouse spinal cord mRNAs and found upregulation of Sox2 expression, a transcription factor that regulates self-renewal and potency of embryonic neural stem cells, in adult SCI in both oligodendrocyte and ependymal cells of the central canal [45].

With application to amyotrophic lateral sclerosis (ALS), Dodge, et al. showed that expression of adeno-associated virus serotype 4 (AAV4)-mediated expression of insulin-like growth factor-1 (IGF-1) or vascular endothelial growth factor (VEGF-165) found in the ventricular system, including the ependymal cell layer and spinal cord central canal, lead to trophic factor delivery throughout the central nervous system and increased survival of cytosolic copper–zinc superoxide dismutase (SOD1) in transgenic mice [46]. The most frequent cause of familial ALS is due to a mutation in the gene encoding SOD1, and these investigative findings pave the way for the potential treatment of ALS [46]. 

## Conclusions

The human central canal of the spinal cord has been studied infrequently. A better understanding of its normal and pathological anatomy is necessary for clinicians who treat or interpret imaging of the spinal cord.
